# Modeling *ANKRD26* 5′-UTR mutation-related thrombocytopenia

**DOI:** 10.1242/dmm.052222

**Published:** 2025-04-28

**Authors:** Liang Zheng, Zhijian Wu, Noritaka Yada, Szumam Liu, Cindy Lin, Antonia Bignotti, Xinyang Zhao, X. Long Zheng

**Affiliations:** ^1^ Department of Pathology and Laboratory Medicine, The University of Kansas Medical Center, Kansas City, KS 66126, USA; ^2^ Institute of Reproductive and Developmental Sciences, The University of Kansas Medical Center, Kansas City, KS 66126, USA; ^3^ Department of Pulmonary and Critical Care Medicine, The Second Xiangya Hospital, Central South University, Changsha 410010, China

**Keywords:** ANKRD26, Thrombocytopenia, Inflammation, Thrombosis, TTP

## Abstract

Mutations in the 5′-untranslated region (5′-UTR) of ankyrin repeat domain-containing protein 26 (*ANKRD26*) are associated with hereditary thrombocytopenia 2 (THC2). However, the causative role of these mutations and the mechanisms underlying THC2 are not fully established. Here, we report, for the first time, that zebrafish carrying a deletion of two nucleotides (Δ2) in the 5′-UTR of *ankrd26* recapitulate some of the key laboratory features of THC2*. ankrd26^ku6^* (homozygous for the Δ2 deletion in the 5′-UTR) fish larvae exhibited significantly increased expression of *ankrd26* mRNA and protein. Adult *ankrd26^ku6^* fish exhibited spontaneous thrombocytopenia. Furthermore, the thrombocytes from *ankrd26^ku6^* fish showed enhanced ability to adhere and aggregate on a collagen surface under flow. Proteomic profiling demonstrated marked upregulation of Ninjurin 1 in young thrombocytes from *ankrd26^ku6^* fish compared with those from wild-type controls. The *ankrd26^ku6^* fish with a homozygous *nacre* allele developed myelodysplastic syndrome at old age. ANKRD26 protein levels were also significantly increased in platelets and plasma from patients with immune thrombotic thrombocytopenic purpura compared with those from unaffected controls. We conclude that ANKRD26 overexpression, resulting from either hereditary or acquired mechanisms, contributes to thrombocytopenia, thrombosis and hematologic malignancies.

## Teaser

**Summary:** A small deletion mutation in the 5'-UTR of *ankrd26* results in spontaneous thrombocytopenia in zebrafish, recapitulating the phenotype in patients with thrombocytopenia 2.

## INTRODUCTION

Thrombocytopenia 2 (THC2) is a rare inherited thrombocytopenia, typically associated with mutations in the 5′-untranslated region (5′-UTR) of ankyrin repeat domain-containing protein 26 (*ANKRD26*) (Pippucci et al., 2011). Patients with THC2 may present with mild bleeding due to life-long thrombocytopenia. These patients may have an increased risk of developing myeloid malignancies, including myelodysplastic syndrome, acute myelogenous leukemia (AML) and chronic myelogenous leukemia (Botero et al., 2015; Kewan et al., 2020; Perez Botero et al., 1993). In previous studies, ∼9% of patients with THC2 developed AML (Noris et al., 2011), or four of 28 family members were found to have AML or myelodysplastic syndrome (Marquez et al., 2014). This suggests that mutations in the 5′-UTR of *ANKRD26* alone are not sufficient to cause malignancy transformation, and other genetic abnormalities or environmental factors are needed to trigger the development of hematological malignancy.

The diagnosis of THC2 in adults can be challenging. Many patients were initially diagnosed for immune thrombocytopenia and received an ineffective or potentially harmful treatment (Averina et al., 2017). To date, there is no specific treatment available for THC2 except for supportive therapy, such as the use of hemostatic agents for patients with bleeding or requiring a major surgical procedure. Platelet transfusions are reserved for patients with severe bleeding or performing procedures with a high risk of bleeding (Balduini et al., 2013; Perez Botero et al., 1993).

ANKRD family proteins contain at least one N-terminal ankyrin (ANK) domain, comprising ∼33 amino acids as an L-shaped structure of one beta-hairpin and two alpha-helices (Mosavi et al., 2004). The biological functions of ANKRD proteins remain largely unknown (Parra et al., 2015). ANKRD17 has been shown to contribute to DNA replication (Deng et al., 2009), vascular development (Hou et al., 2009), and inflammatory responses (Menning and Kufer, 2013). ANKRD26 may mediate protein–protein interactions and activate an intracellular signaling pathway (Liu et al., 2012). It is abundantly expressed in various tissues, including the brain, liver, adipose tissue, skeletal muscle and hematopoietic tissues (Raciti et al., 2011). Previous studies demonstrated that *Ankrd26*-deficient mice develop extreme obesity, insulin resistance and gigantism (Acs et al., 2015; Bera et al., 2008; Raciti et al., 2011). In humans, mutations in 5′-UTR of *ANKRD26* have been implicated in the pathogenesis of THC2 (Husnain et al., 2019; Noris et al., 2013, 2011; Perez Botero et al., 1993; Pippucci et al., 2011), although no causative role has been demonstrated in animal models. *ANKRD36*, a homolog of *ANKRD26*, is implicated in the pathogenesis of hypertension (Yan et al., 2021) and type 2 diabetes mellitus, and it may also serve as a biomarker of chronic inflammation (Fang et al., 2018). Mutations in *ANKRD36* have been found to be associated with immune thrombotic thrombocytopenic purpura (iTTP) (Basu et al., 2021), although the underlying mechanism is unknown.

Most patients with THC2 harbor a point substitution or a small (1-2 bp) deletion in the 5′-UTR of *ANKRD26* (Kewan et al., 2020) with a dominant inheritance. These mutations are mainly located at the runt-related transcription factor 1 (RUNX1)/friend leukemia integration factor 1 (FLI1) bind site or adjective area (Bluteau et al., 2014; Kewan et al., 2020). It was suggested that the RUNX1/FLI1 complex negatively regulates *ANKRD26* expression and that mutations in 5′-UTR of *ANKRD26* disrupt RUNX1/FLI1 inhibition, which would lead to overexpression of ANKRD26 in megakaryocytes and defective proplatelet formation, and thus thrombocytopenia in patients (Balduini et al., 2018; Bluteau et al., 2014). However, many mutations found in patients with THC2 are not located in the RUNX1/FLI1 binding region, suggesting that additional mechanisms contribute to the pathogenesis of THC2.

Here, using CRISPR/Cas9, we generated the first zebrafish model of THC2 by introducing a small deletion mutation in the 5′-UTR of *ankrd26* (also known as *si:ch211-272n13.3*). The *ankrd26* mutant zebrafish showed overexpression of *ankrd26* at mRNA and protein levels. Most importantly, the homozygous mutant zebrafish exhibited spontaneous thrombocytopenia, recapitulating the disease phenotype in human patients with THC2. Mass spectrometric analysis identified increased levels of Ninjurin 1 (Ninj1) in thrombocytes that might contribute to enhanced inflammation and thrombosis. Our findings could help develop a targeted therapeutic for ANKRD26-related thrombocytopenia and hematologic malignancy.

## RESULTS

### Generation and characterization of *ankrd26* mutant zebrafish

**Fig. 1.**
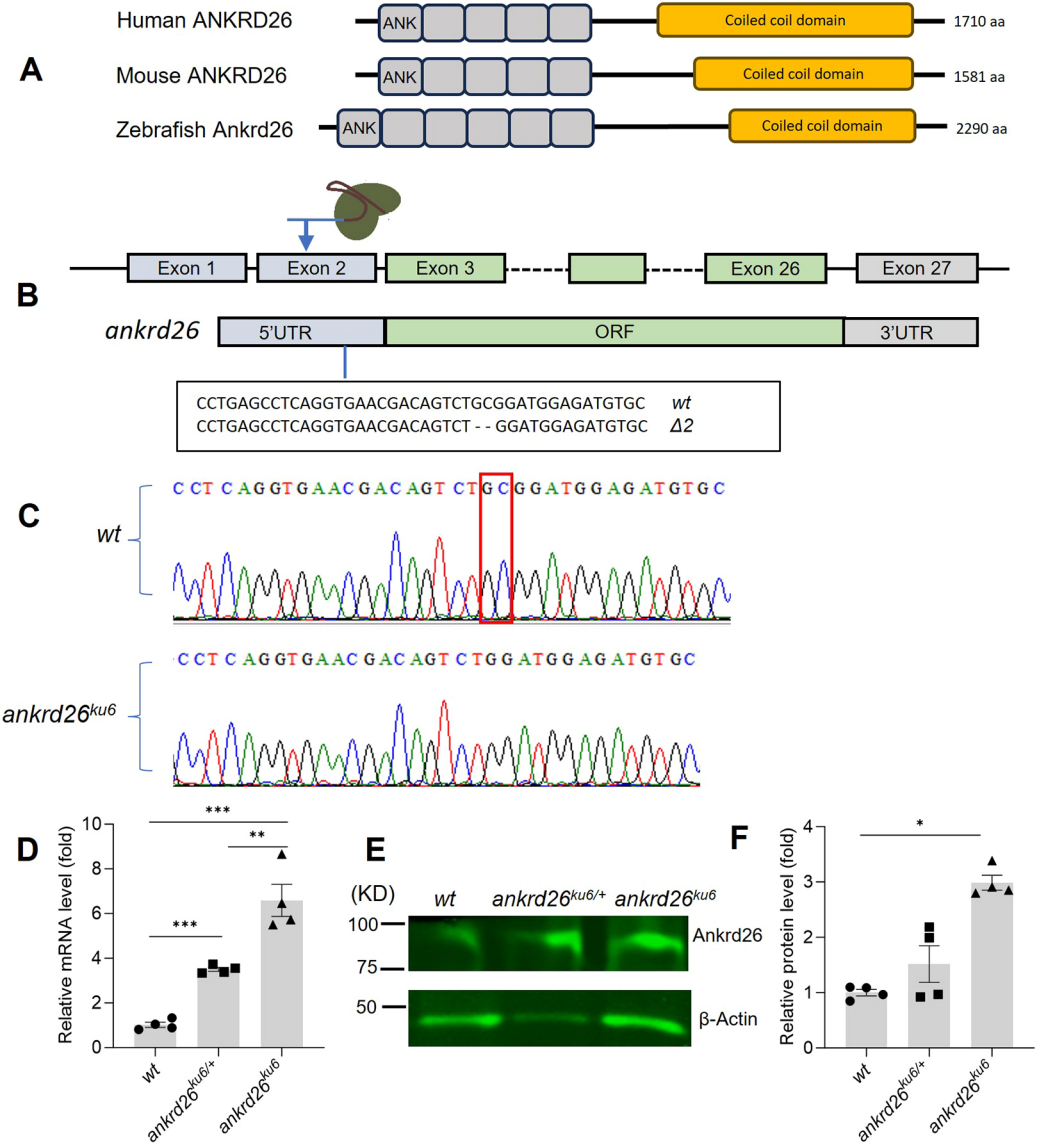
**Generation and characterization of *ankrd26^ku6^* zebrafish.** (A) Schematic representation of human, mouse and zebrafish Ankrd26 protein, comprising N-terminal ankyrin (ANK) modules and C-terminal coiled-coil domain. (B) Schematic representation of the genomic DNA structure of *ankrd26* and its encoded mRNA, showing the targeted area in the 5′-untranslated region (5′-UTR) of *ankrd26* by CRISPR/Cas9 and the two deleted nucleotides (nt) in the 5′-UTR (Δ2). ORF, open reading frame. (C) Sanger sequencing confirmed the deletion of a two-nucleotide (GC) sequence (boxed). This mutant allele was named as *ku6* in ZFIN. (D) Relative *ankrd26* mRNA levels in pooled wild-type (wt), *ankrd26^ku6/+^* (heterozygote) and *ankrd26^ku6^* (homozygote) zebrafish larvae (*n*=10) at 5 days post-fertilization (dpf). (E,F) Western blotting (E) and densitometric analysis (F) of Ankrd26 protein expression levels in wt, *ankrd26^ku6/+^* and *ankrd26^ku6^* zebrafish larvae. β-Actin protein was used as protein loading control in E. Data are means±s.e.m. of at least three independent experiments. Mann–Whitney *U*-test was used to analyze differences between two groups. *P<0.05, **P<0.01 and ***P<0.005.

Sequence analysis and structural predictions revealed that human and murine ANKRD26 proteins contain five ankyrin repeats at the N-terminus and a large coiled-coil domain at the C-terminus. The domain structure of zebrafish Ankrd26 is similar to that of human ANKRD26, with six predicted ankyrin repeats and amino acid sequence similarity of 48%. To determine the biological functions of *ankrd26*, we used CRISPR/Cas9 to generate a small deletion mutation in exon 2, corresponding to the area in 5′-UTR of zebrafish *ankrd26* (Fig. 1A). The founder mutant fish were crossed with wild-type (wt) zebrafish to produce heterozygous offspring carrying the desired mutation. The selected heterozygous zebrafish were then crossed with wt fish for three generations to reduce potential off-target effects from Cas9. One mutant line carrying a small (2 bp) deletion (Δ2) in the 5′-UTR of *ankrd26* was chosen for further characterization (Fig. 1B,C). Real-time quantitative polymerase chain reaction (qPCR) analysis demonstrated ∼3.5-fold and ∼7.0-fold increased levels of *ankrd26* mRNA in the heterozygous (*P*<0.005) and homozygous (*P*<0.005) mutant larvae, respectively, compared to those in the wt controls (Fig. 1D). Western blotting confirmed significantly increased levels of Ankrd26 protein in homozygous (*ankrd26^ku6^*) fish larvae compared with those in the wt control larvae (Fig. 1E,F). These results indicate that the mutation in the 5′-UTR of *ankrd26* results in increased expression of *ankrd26* mRNA and protein in zebrafish.

**Fig. 2.**
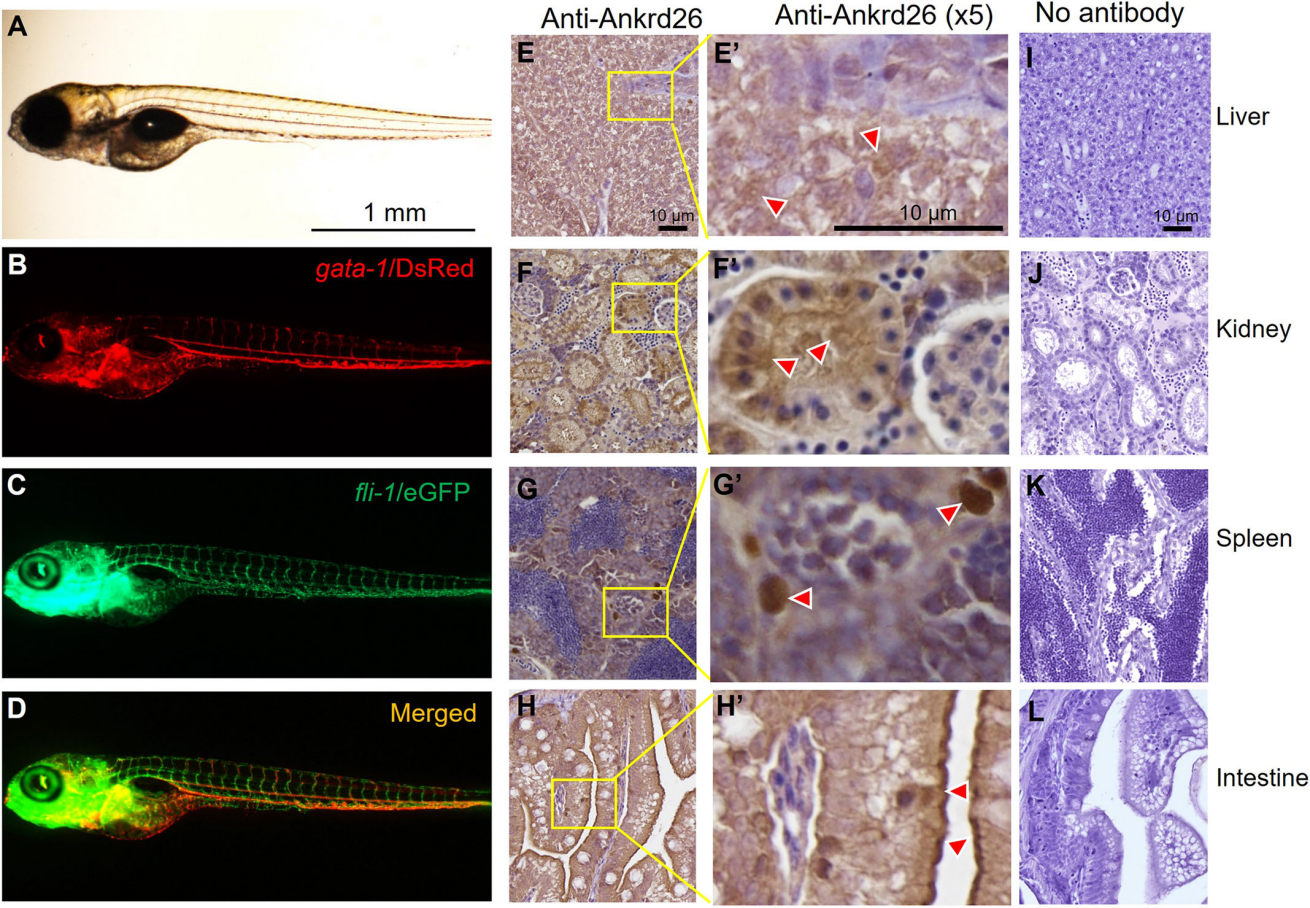
**Morphology of zebrafish larvae and immunohistochemical detection of Ankrd26 protein in adult zebrafish tissues.** (A-D) Representative morphology of a 4 dpf larva, including a light microscopic image, a red fluorescent (*gata*-1/DsRed) image, a green fluorescent (*fli*-1/eGFP) image and a merged image as indicated. (E-H) Immunohistochemical staining of Ankrd26 protein in hepatocytes in the liver (E,E′), proximal or distal tubules in the kidney (F,F′), ellipsoids in the spleen (G,G′) and epithelial cells in the intestinal villi (H,H′). Red arrowheads indicate representative Ankrd26-postivie cells. (I-L) Negative controls for immunohistochemical staining in the liver (I), kidney (J), spleen (K) and intestine (L), without incubation with primary antibody against zebrafish Ankrd26.

*ankrd26* mutant fish exhibited normal gross morphology, indistinguishable from that of wt fish under light and fluorescent microscopes (Fig. 2A-D). Immunohistochemistry on fixed adult zebrafish organ tissues demonstrated widely expressed Ankrd26 protein in various zebrafish tissues, particularly in the liver hepatocytes (Fig. 2E,E′), renal proximal and distal tubular cells (Fig. 2F,F′), splenic ellipsoids (Fig. 2G,G′) and epithelial cells of the intestinal villi (Fig. 2H,H′). No staining was observed in tissue sections in which the primary antibody against Ankrd26 was omitted (Fig. 2I-L), suggesting that the staining for Anrkd26 in these tissues was specific.

### Homozygous *ankrd26^ku6^* zebrafish develop spontaneous thrombocytopenia

**Fig. 3.**
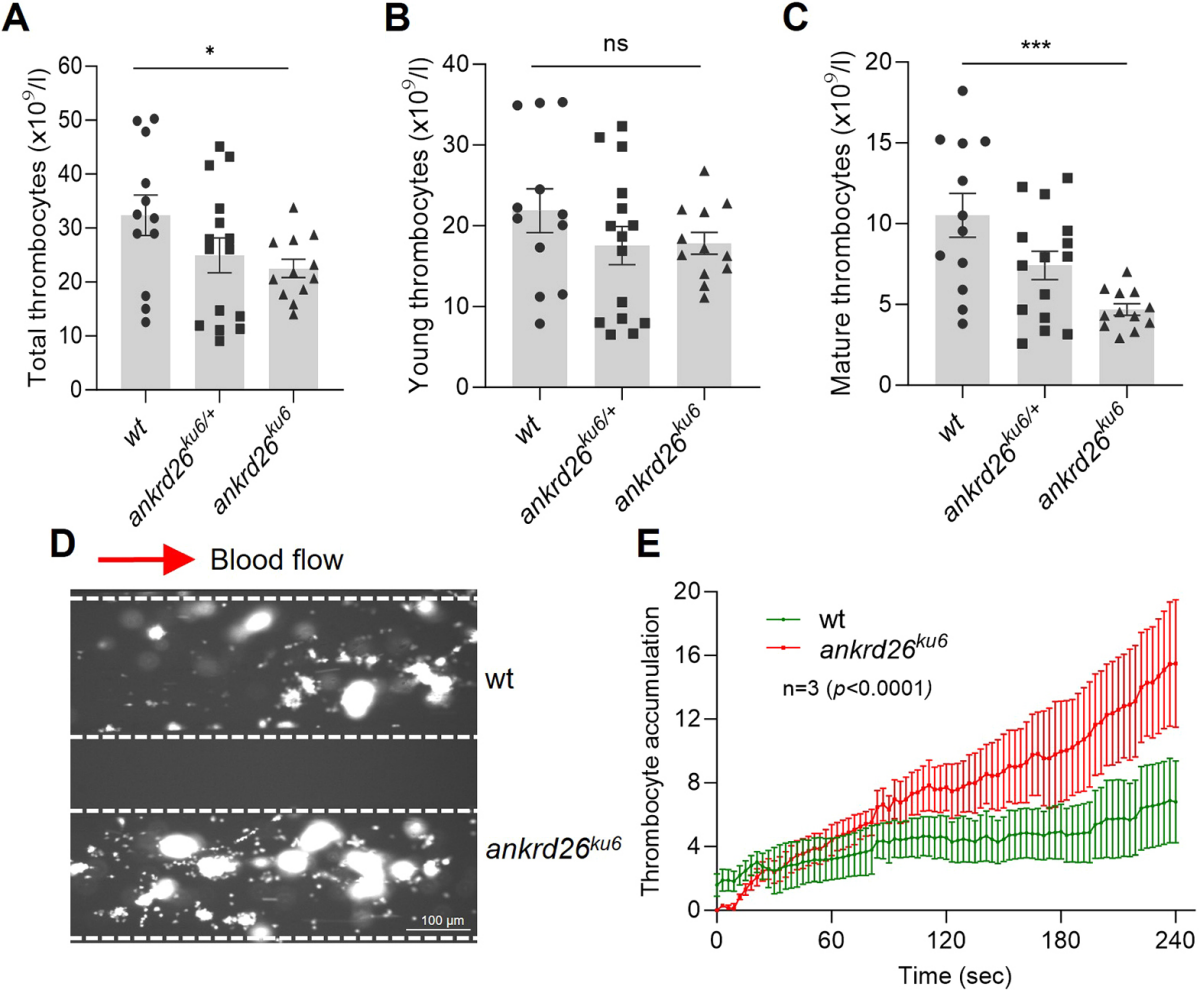
**Thrombocyte counts and their adhesion/aggregation on a collagen surface under flow.** (A-C) Total (A), young (B) and mature (C) thrombocyte counts in wt (*n*=12), *ankrd26^ku6/+^* (*n*=15) and *ankrd26^ku6^* (*n*=12) zebrafish. The data shown represent the individual values, mean and s.e.m. Kruskal–Wallis analysis was used to determine statistical significance. (D) The surface coverage of fluorescent thrombocytes on a fibrillar collagen-coated surface in the microfluidic channel after perfusion of pooled whole blood obtained from wt (top) and *ankrd26^ku6^* (bottom) zebrafish under arterial shear (15 dyne/cm^2^). (E) The rate of fluorescence accumulation (or thrombocyte adhesion) on a fibrillar collagen-coated surface following perfusion of pooled whole blood from wt and *ankrd26^ku6^* zebrafish. Data are presented as the mean±s.e.m. from three independent experiments. ns, *P*>0.05; *P<0.05 and ***P<0.005.

Taking advantage of an established transgenic zebrafish line that expresses red fluorescent protein under the *gata-1* (also known as *gata1a*) promoter (for erythrocytes and young thrombocytes) and green fluorescent protein under the *fli1* promoter (for young and mature thrombocytes) (Zheng et al., 2020), we determined the number of total, young, and mature thrombocytes by flow cytometry. As shown, there was a statistically significant reduction in total thrombocyte counts (mean±s.e.m.) (22.5±5.8×10^9^/l) (Fig. 3A) in the *ankrd26^ku6^* zebrafish compared to those in the wt zebrafish (32.4±12.9×10^9^/l). Such a reduction in total thrombocyte counts was not the result of a decrease in young thrombocyte count (*P*>0.05) (Fig. 3B) but in mature thrombocyte count (*P*<0.001) (Fig. 3C). These results suggest that homozygous mutations in the 5′-UTR of *ankrd26* cause thrombocytopenia, likely resulting from impaired thrombocyte maturation.

### *ankrd26^ku6^* thrombocytes exhibit enhanced adhesion/aggregation onto a collagen surface under shear

To determine whether mutations in the 5′-UTR of *ankrd26* modulate thrombus potential, we performed a shear-dependent thrombocyte adhesion and aggregation assay using a microfluidic system. When D-phenylalanyl-L-prolyl-L-arginine chloromethyl ketone (PPACK)-anticoagulated whole-blood samples were perfused over fibrillar collagen-coated surfaces under 15 dyne/cm^2^ shear, the rate of thrombocyte adhesion and aggregation (Fig. 3D), and the surface thrombocyte coverage at the end of perfusion (Fig. 3E) on the collagen surfaces were significantly increased in the blood collected from *ankrd26^ku6^* zebrafish compared to that from the wt controls, despite significantly lower thrombocyte counts in the *ankrd26^ku6^* zebrafish. These results indicate that thrombocytes or other components in the blood of *ankrd26^ku6^* zebrafish are more thrombogenic than those in the blood of wt controls.

### *ankrd26^ku6^* zebrafish demonstrate a higher mortality rate on the *nacre* background

**Fig. 4.**
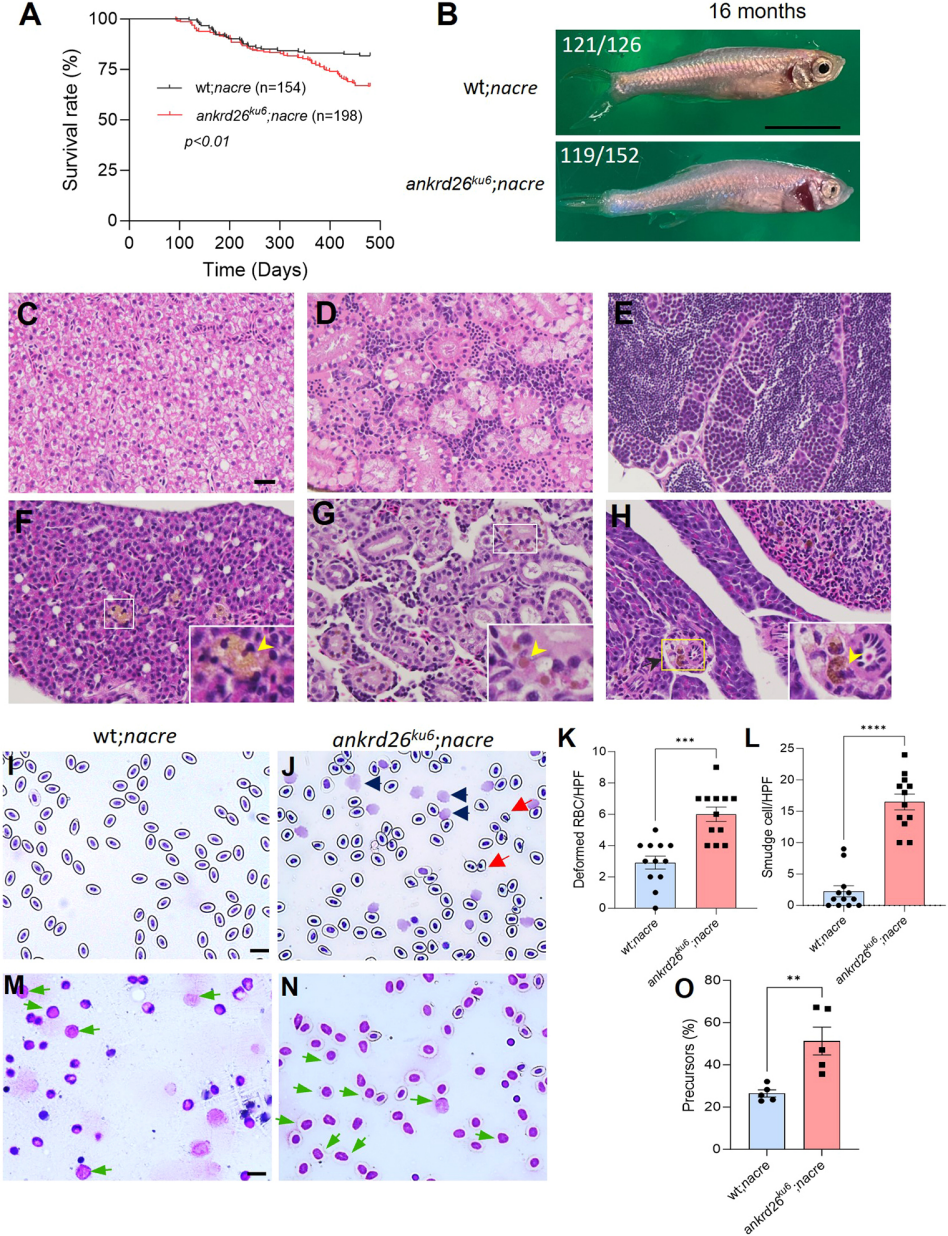
**Survival and histological analyses of tissues, blood and kidney marrow of zebrafish of various genotypes.** (A) Kaplan–Meier survival analysis indicates the survival rates of wt*;nacre* (*n*=154) and *ankrd26^ku6^;nacre* (*n*=198) zebrafish over a 16-month observation. (B) Representative gross morphology in wt*;nacre* and *ankrd26^ku6^;nacre* zebrafish at the age of 16 months. The proportions of abnormal body shapes are indicated at the top left in images. (C-H) Hematoxylin and Eosin staining indicates the absence (C-E) and presence (F-H) of lipofuscin deposition in wt*;nacre* and *ankrd26^ku6^;nacre* zebrafish, respectively, at the age of 16 months, in the liver (C,F), kidney (D,G) and spleen (E,H). Yellow arrowheads indicate representative lipofuscin deposition in the organ tissues of *ankrd26^ku6^;nacre* zebrafish. (I,J) Giemsa staining of peripheral blood smears from wt*;nacre* (I) and *ankrd26^ku6^;nacre* (J) zebrafish. Red and black arrowheads in J indicate deformed red blood cells and smudge cells, respectively. (K,L) Quantitation of the deformed red blood cells (K) and smudge cells (L) in peripheral blood smears of wt*;nacre* and *ankrd26^ku6^;nacre* zebrafish. HPF, high-power field; RBC, red blood cells. (M,N) Representative images of stained kidney marrow cells in wt*;nacre* (M) and *ankrd26^ku6^;nacre* (N) zebrafish. Green arrows indicate the increased number of myeloid progenitor cells in *ankrd26^ku6^* zebrafish. (O) Quantitation of myeloid progenitor cells in kidney marrow smears from wt*;nacre* and *ankrd26^ku6^;nacre* zebrafish at the age of 16 months. Data in K, L and O are individuals, means (bars) and s.e.m. (horizontal lines). Mann–Whitney *U*-test was performed to determine the statistical significance of differences between the two groups. **P<0.01, ***P<0.005, and ****P<0.0001. Scale bars: 10 µm (C,I,M); 1 cm (B).

To better observe the morphological changes (particularly blood vessels and blood clots) in the live zebrafish, we outcrossed the *ankrd26^ku6^* zebrafish with a transparent zebrafish line, homozygous for *nacre*. The *nacre* allele corresponds to a mutation in the *mitfa* gene, which disrupts melanophore development, resulting in unpigmented skin and scales in zebrafish (Lister et al., 1999). Kaplan–Meier survival analysis revealed a modest, but statistically significant, increase in mortality rate in the *ankrd26^ku6^;nacre* zebrafish (*n*=198) compared to that in the wt controls (*n*=154). At the age of 16 months (old age), the survival rates in the *ankrd26^ku6^;nacre* group and control group were 66.7% and 81.8%, respectively (*P*<0.01) (Fig. 4A). Furthermore, 78.2% (119/152) of *ankrd26^ku6^* zebrafish on the *nacre* background at the age of 16 months displayed an abnormal gross morphology (e.g. pale in color and curved in body shape) compared with 3.9% (5/126) of the wt controls (Fig. 4B), suggesting the presence of severe body stress in *ankrd26^ku6^* zebrafish.

Histological analysis revealed significant deposition of lipofuscin, a brownish pigment resulting from damaged blood cells, in major organs, including the liver, kidneys and spleen of *ankrd26^ku6^;nacre* zebrafish (Fig. 4F-H), but not in the wt*;nacre* controls at the same age (Fig. 4C-E), suggesting chronic blood cell stress, broken blood cells and absorption of the byproducts in these tissues (Hoda and Hoda, 2020). Further morphological analysis of peripheral blood cells demonstrated increased numbers of broken red blood cells and smudge cells in *ankrd26^ku6^;nacre* zebrafish compared with those in wt*;nacre* controls at the age of 16 months (Fig. 4I-L). Finally, morphological analysis of zebrafish kidney marrow cells, the function equivalent cells of human bone marrow, revealed a significantly increased percentage of precursor cells in the a*nkrd26^ku6^;nacre* zebrafish compared with that in the wt*;nacre* controls (Fig. 4M-O). Such malignant transformation was not observed in the wt and *ankrd26^ku6^* zebrafish without *nacre* mutation. These findings suggest that aged zebrafish carrying a homozygous mutation in the 5′-UTR of *ankrd26* (*ankrd26^ku6^*) on top of *nacre* have increased susceptibility to developing hematological malignancy.

### Proteomic analysis identifies upregulation of Ninj1 in *ankrd26^ku6^* thrombocytes

**Fig. 5.**
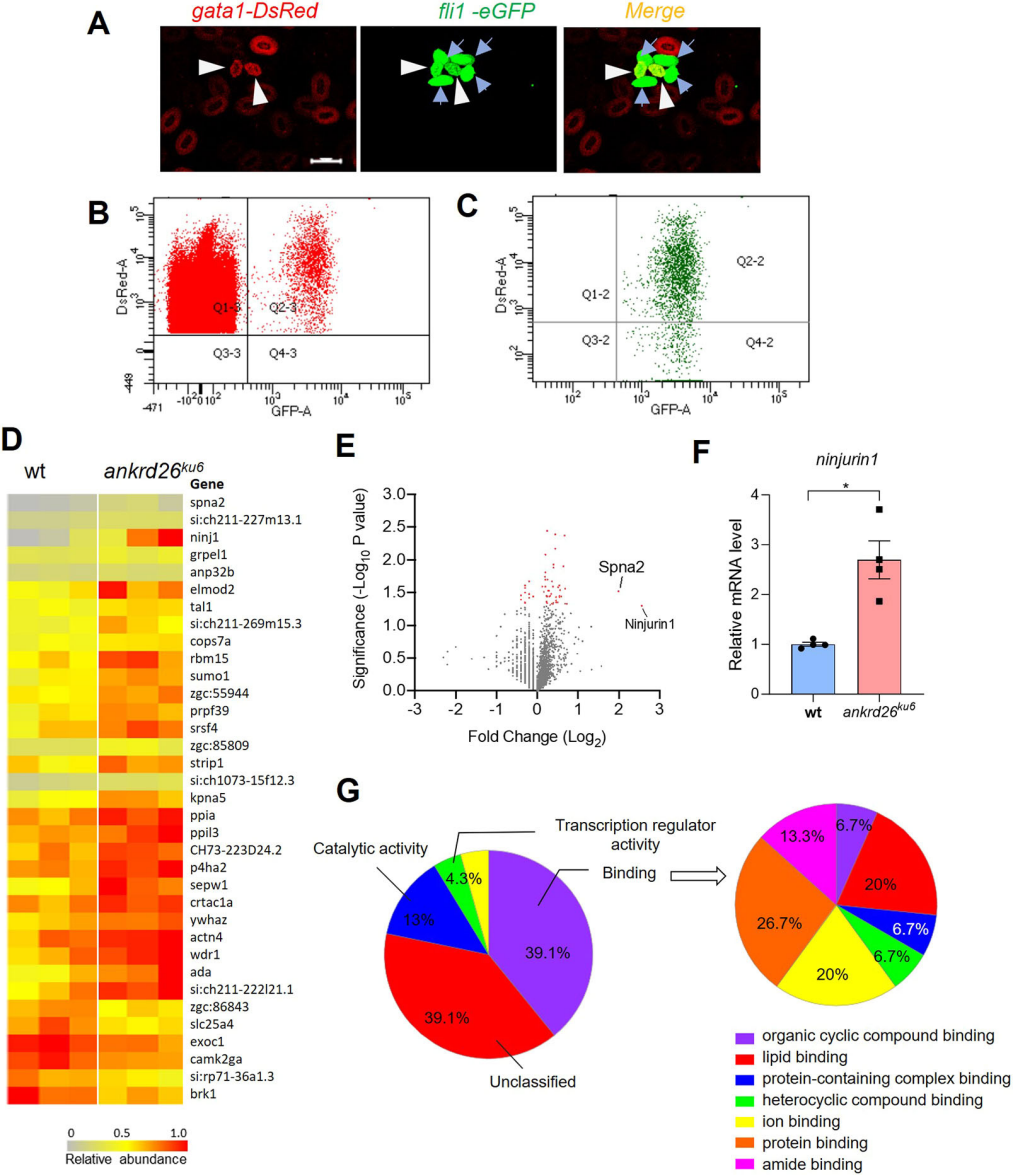
**Differential proteomics in young thrombocytes of wt and *ankrd26^ku6^* zebrafish.** (A) Endogenous fluorescence labeling to differentiate erythrocytes (only in red), young thrombocytes (in both red and green as indicated by white arrowheads) and mature thrombocytes (only in green as indicated by blue arrows). (B) Flow cytometric gating for erythrocytes (Q1-3) and thrombocytes (Q2-3). (C) Purity of fluorescence-activated cell sorting-isolated young (Q2-2) and mature (Q4-2) thrombocytes. (D) Heat map showing the relative abundance of 35 ranked proteins identified in young thrombocytes of wt and *ankrd26^ku6^* zebrafish. Ranking is based on fold change >1.25 (*n*=3). (E) Volcano plot demonstrating the fold change of 2425 proteins identified in young thrombocytes of wt and *ankrd26^ku6^* zebrafish (*n*=3). Red dots represent 35 differentially expressed proteins with fold change >1.25 (*P*<0.05). (F) Quantification of *ninjurin 1* mRNA levels in zebrafish larvae of different genotypes. (G) Molecular function of proteins with significant change in their expression in young thrombocytes of *ankrd26^ku6^* zebrafish compared with those of wt controls.

The underlying mechanisms of *ANKRD26* mutation-associated thrombocytopenia remain largely unexplored, apart from evidence suggesting overactivation of the mitogen-activated protein kinase (MAPK)/extracellular signal-regulated kinase (ERK) signaling pathway in *in vitro*-cultured megakaryocytes from patients with THC2 (Bluteau et al., 2014). To further understand the molecular basis of *ANKRD26-*associated thrombocytopenia, we performed mass spectrometric analysis of fluorescence-activated cell sorting (FACS)-isolated thrombocytes (mature versus young) (Fig. 5A-C) from zebrafish of different genotypes. Our results showed that 2524 proteins were identified in isolated thrombocytes. Of these, 35 proteins exhibited more than 1.25-fold change in young thrombocytes from *ankrd26^ku6^* zebrafish compared with those from the wt controls (*P*<0.05; *n*=3) (Fig. 5D; Table S1). The most upregulated protein (5.9-fold) found in young thrombocytes of *ankrd26^ku6^* zebrafish was Ninj1 (Fig. 5E). Real-time qPCR confirmed the increase (∼3-fold) in *ninj1* mRNA in *ankrd26^ku6^* fish larvae compared with that in the wt controls (*P*<0.05) (Fig. 5F). Gene Ontology (GO) analysis demonstrated that the molecular functions are associated with differentially expressed proteins in the young thrombocytes of *ankrd26^ku6^* zebrafish (Fig. 5G). The most enriched molecular function is linked to binding activities, with the predominant categories being protein, ion and lipid binding, suggesting the physiological roles of Ankrd26 in protein–protein interactions, ion regulation and lipid-associated activities within the young thrombocytes.

**Fig. 6.**
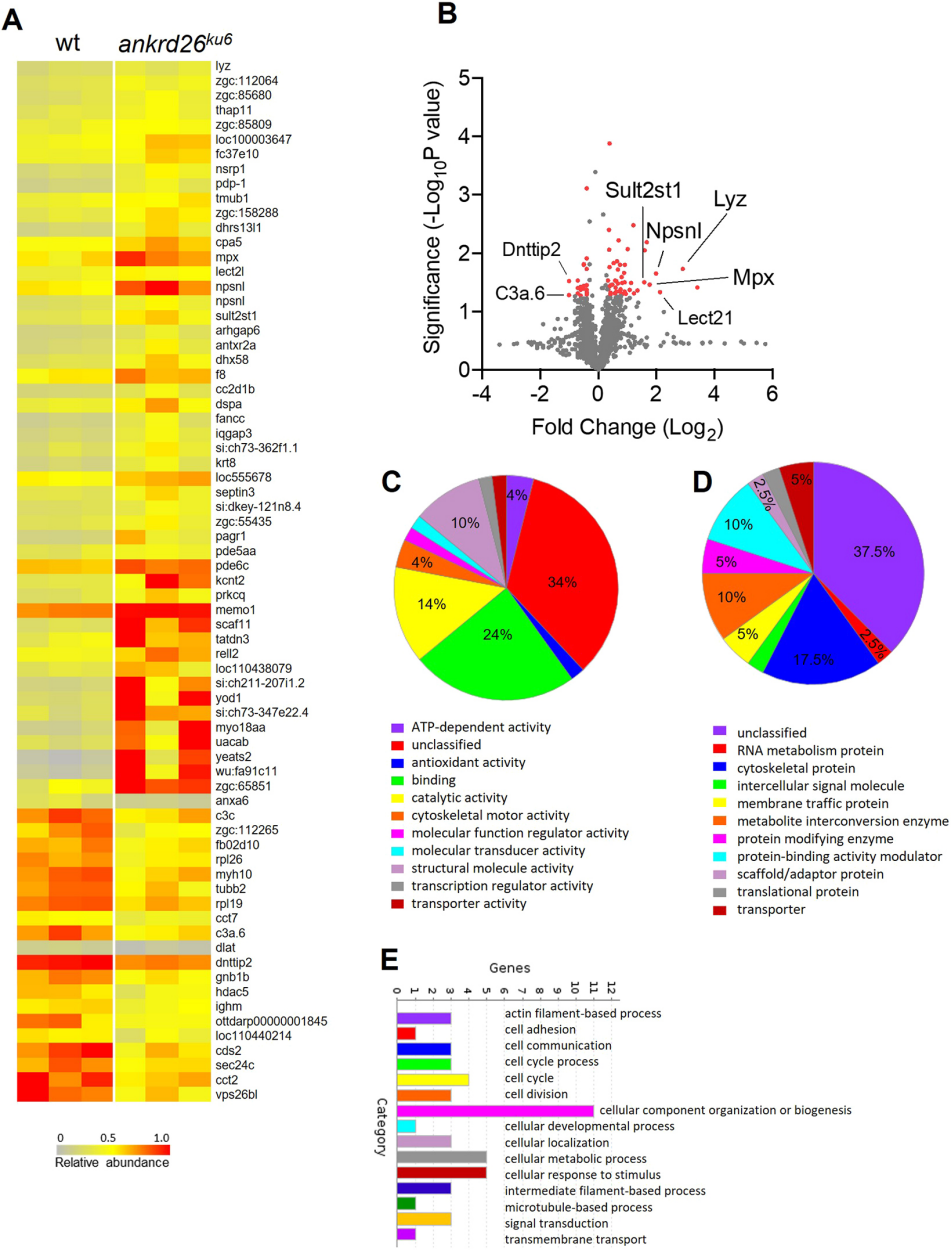
**Differential proteomics in mature thrombocytes from wt and *ankrd26^ku6^* zebrafish.** (A) Heat map showing the 71 differentially expressed proteins identified in the mature thrombocytes from wt and *ankrd26^ku6^* zebrafish. Ranking is based on fold change >1.25 (*n*=3). (B) Volcano plot demonstrating the fold change of 2425 proteins in mature thrombocytes from *ankrd26^ku6^* zebrafish (*n*=3) compared with those from wt controls (*n*=3). Each sample represents pooled blood from ten individual zebrafish. Red dots represent 71 differentially expressed proteins with significant fold change >1.25 (*P*<0.05). (C-E) Molecular functions (C), classes (D) and cellular processes (E) of proteins with significantly changed expression in mature thrombocytes of *ankrd26^ku6^* zebrafish compared with those of wt controls.

Furthermore, in a comparison of protein expression in the mature thrombocytes between *ankrd26^ku6^* zebrafish and wt controls, 71 of 2524 identified proteins exhibited a greater than 1.25-fold change (*P*<0.05; *n*=3) (Fig. 6A; Table S2). The most upregulated proteins were Lysozyme (Lyz), Leukocyte cell-derived chemotaxin 2 like (Lect2l; also known as Lect2.1), Metalloendopeptidase (Npsnl; also known as Npsn), Eosinophil peroxidase isoform X1 (Mpx) and Sulfotransferase (Sult2st1). Several proteins including Deoxy-nucleotidyl-transferase terminal interacting protein 2 (Dnttip2) and Complement c3-h1-like precursor (C3a.6) were significantly downregulated (Fig. 6B). Exploring the molecular functions associated with these altered proteins in *ankrd26^ku6^* mature thrombocytes and GO analysis also revealed significant enrichment in binding activities, like that in young thrombocytes (Fig. 6C). Furthermore, when categorizing these proteins based on class, cytoskeletal proteins were most enriched, followed by metabolite interconversion enzymes and protein-binding activity modulators (Fig. 6D), consistent with their enhanced adhesion and aggregation activity. Notably, the most enriched cellular process was identified as cellular component organization or biogenesis (Fig. 6E). These results support a hypothesis that potential disruptions in the structural and organizational integrity of mature thrombocytes result in altered survival of thrombocytes in the circulation.

### ANKRD26 might be a phenotypic modifier in thrombotic thrombocytopenic purpura

**Fig. 7.**
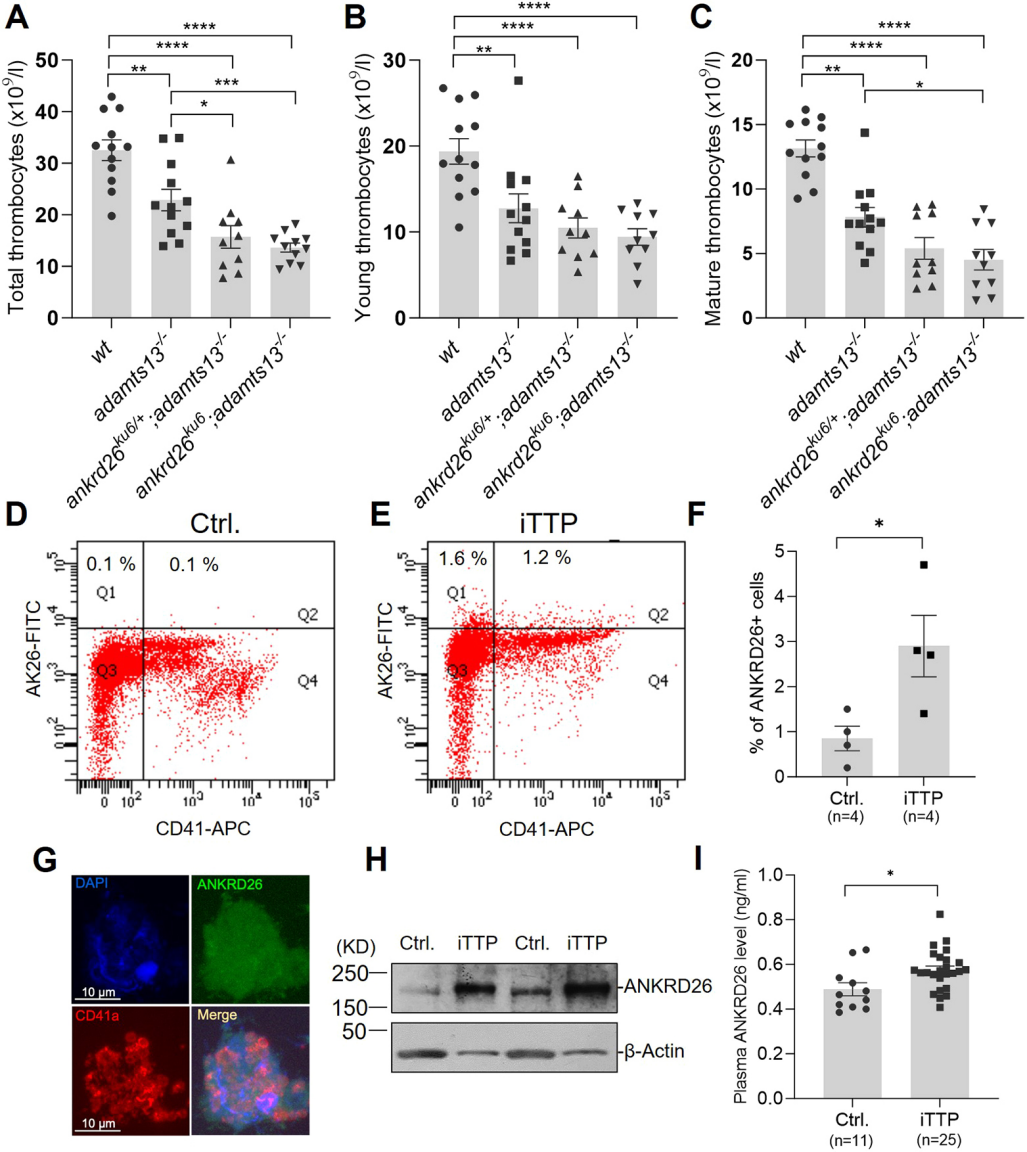
**ANKRD26 is a potential modifier of the thrombotic thrombocytopenic purpura phenotype.** (A-C) Total (A), young (B) and mature (C) thrombocyte counts in zebrafish (6-8 months old) of different genotypes as indicated. Data are presented as individual values, mean (bars) and s.e.m. (horizontal lines). Kruskal–Wallis analysis was performed to determine statistical significance. (D,E) Flow cytometric analysis showing the percentage of ANKRD26-positive (Q1+Q2) cells in the peripheral blood buffy coat from unaffected controls (Ctrl.; D) and patients with immune thrombotic thrombocytopenic purpura (iTTP; E). (F) Quantification of ANKRD26-positive cells in the controls (*n*=4) and patients with iTTP (*n*=4). (G) Confocal fluorescent imaging showing the expression of ANKRD26 (green) and glycoprotein 1bα protein (CD41a; red) in megakaryocytes from peripheral blood smears of patients with iTTP. The nucleus was stained with DAPI (blue). (H) Western blot analysis demonstrating the levels of ANKRD26 protein in platelet lysates of controls and patients with iTTP. (I) Quantitation of plasma levels of soluble ANKRD26 protein in unaffected controls (*n*=11) and patients with iTTP (*n*=25). *P<0.05, **P<0.01, ***P<0.005, and ****P<0.0001.

The only clue linking ANKRD26 to pathogenesis of thrombotic thrombocytopenic purpura (TTP) is the higher frequency of mutations in the ANKRD family identified in patients with iTTP than in unaffected controls (Basu et al., 2021). Because *ankrd26^ku6^* thrombocytes were more thrombogenic than wt thrombocytes, we asked whether ANKRD26 overexpression alters TTP phenotypes. To assess this, we crossed the *ankrd26^ku6^* zebrafish with *adamts13^−/−^* zebrafish to generate offspring carrying double mutations. Our results showed that homozygous (*ankrd26^ku6^*) or heterozygous (*ankrd26^ku6/+^*) zebrafish on *adamts13^−/−^* background exhibited more severe thrombocytopenia than those carrying a single gene mutation alone. Severe thrombocytopenia, defined by total thrombocyte count <15×10^9^/l, occurred in 45.4% and 63.6% of *ankrd26^ku6/+^;adamts13^−/−^* and *ankrd26^ku6^;adamts13^−/−^* zebrafish, respectively, compared to 0% of the wt or *ankrd26^ku6/+^* zebrafish (Fig. 7A). The reduction in mature thrombocyte counts (mean±s.e.m.) was even more profound in the zebrafish with double gene mutations (5.4±2.6×10^9^/l for *ankrd26^ku6/+^;adamts13^−/−^*) than in a*damts13^−/−^* (7.8±2.6×10^9^/l) (Fig. 7B,C) or *ankrd26^ku6/+^* (7.4±3.4×10^9^/l) zebrafish (Fig. 3C). These findings indicate that mutation in the 5′-UTR of *ankrd26* that results in overexpression of Ankrd26 protein may modify the phenotype of TTP or other thrombotic microangiopathic disorders.

### Increased cellular and plasma ANKRD26 protein levels in patients with iTTP

To further gain insight into the pathophysiological role of ANKRD26 in iTTP, we determined the levels of cellular and plasma ANKRD26 protein in patients with acute iTTP. First, flow cytometric analysis demonstrated that the percentage of ANKRD26-positive cells was significantly higher in the peripheral blood buffy coat, containing primarily platelets and leukocytes, from patients with acute iTTP compared to that from unaffected controls (Fig. 7D-F). Confocal imaging confirmed the expression of ANKRD26 protein in circulating megakaryocytes from the peripheral blood of patients with iTTP (Fig. 7G). Western blot analysis further confirmed the significantly increased expression of ANKRD26 protein in platelet cell lysates from patients with iTTP compared with those from unaffected controls (Fig. 7H). Moreover, plasma levels of ANKRD26 protein, likely released from ruptured cells owing to inflammation and thrombosis, were significantly elevated in patients with acute iTTP compared with those in unaffected controls (*P*<0.05) (Fig. 7I). Together, these results support the hypothesis that overexpression of ANKRD26 protein disrupts platelet production and promotes inflammation and thrombosis. This dysregulation could contribute to phenotypic modifications of TTP and potentially other inflammatory thrombotic disorders.

## DISCUSSION

This study is the first to demonstrate that a mutation in the 5'-UTR of *ankrd26* leads to spontaneous thrombocytopenia, effectively recapitulating some of the key laboratory features of THC2. Our study also reveals that zebrafish carrying a homozygous mutation in the 5'-UTR of *ankrd26* on top of *nacre* mutation develop hematological malignancy, which is not observed in *ankrd26^ku6^* zebrafish without the *nacre* allele. This suggests that *ankrd26^ku6^* alone is insufficient to drive malignant transformation and that the *nacre* allele alters cellular or microenvironmental contexts – possibly through effects on pigmentation pathways, immune function or stem cell regulation – that enable or enhance the oncogenic potential of Ankrd26 overexpression. Additionally, our results demonstrate a potentially synergistic role of Ankrd26 overexpression and severe Adamts13 deficiency in the pathogenesis of TTP in a zebrafish model. We further discovered that Ninj1, a key inflammatory mediator and membrane-rupturing protein, is most significantly upregulated in young thrombocytes of zebrafish carrying the homozygous mutation (*ankrd26^ku6^*) in the 5′-UTR of *ankrd26*. Finally, we demonstrate that ANKRD26 levels were significantly elevated in platelets and plasma of patients with acute iTTP. These findings shed new light on the biology of ANKRD26 and could facilitate the design of novel targeted therapeutics for hereditary and acquired thrombocytopenia, such as iTTP, as well as hematological malignancies associated with abnormal ANKRD26 expression.

The mechanisms underlying *ANKRD26* mutation-associated thrombocytopenia and hematological malignancy are still not fully understood. A previous study demonstrated that the binding of RUNX1 and FLI1 to the 5′-UTR of *ANKRD26* is disrupted in cultured megakaryocytes isolated from patients with THC2, which might halt the suppression of transcription of *ANKRD26*, leading to overexpression of *ANKRD26* and overactivation of MAPK/ERK, resulting in impaired proplatelet production and malignancy transformation (Bluteau et al., 2014). However, such a hypothesis does not account for the overexpression of Anrkd26 observed in *ankrd26^ku6^* zebrafish, as the mutated region in the 5′-UTR lacks known Runx1 or Fli1 transcriptional factor binding sites (Fig. S1).

Among the mutations found in the 5′-UTR of *ANKRD26* in patients with THC2, a subset are located within the putative RUNX1 binding region (Bluteau et al., 2014). No mutation in the FLI1 binding site has been reported in patients with THC2 (Bluteau et al., 2014). Many mutations associated with THC2 are not located in RUNX1 or FLI1 binding regions (Sullivan et al., 2022), implying that there are other potential mechanisms resulting in the overexpression of *ANKRD26*. Mutations in the 5′-UTR of *ANKRD26* might alter the mRNA stem-loop structure, which directly affects RNA folding, structural stability and recognition sites for RNA-binding proteins (Malys and McCarthy, 2011; Malys and Nivinskas, 2009), resulting in ANKRD26 expression change.

The *ankrd26^ku6^* zebrafish with a homozygous *nacre* allele at old age (>16 months) develop hematologic malignancy with a significantly increased number of dysplastic myeloid cells such as fragmentation of erythrocytes and smudgy cells on the peripheral blood smear. The presence of smudgy cells is associated with various hematological disorders including AML (Chang et al., 2016; Nowakowski et al., 2009). Such malignant transformation might explain the increased mortality rate in the *ankrd26^ku6^* zebrafish with a homozygous *nacre* allele compared with that in the wt controls, although the exact cause of death of these fish has yet to be determined.

To gain further insight into the mechanisms by which *ankrd26* mutations contribute to thrombocytopenia and potential malignant transformation in zebrafish, we performed quantitative proteomic analysis of isolated thrombocytes. We identified the significant upregulation of Ninj1 expression in young thrombocytes carrying a*nkrd26^ku6^* mutation, compared to that in the wt controls. NINJ1 is a transmembrane adhesion protein that may facilitate cell–cell adhesion through homophilic interactions via its extracellular N-terminal adhesion motifs (Kim et al., 2020). It may also play a role during an inflammatory process through enhancing interactions between immune cells and endothelial cells (Ifergan et al., 2011; Shin et al., 2016; Toma et al., 2020). Furthermore, NINJ1 may contribute to plasma membrane rupture during programmed and necrotic cell death (Kayagaki et al., 2021). Thus, significant increase in Ninj1 expression in young thrombocytes of *ankrd26^ku6^* zebrafish might explain the heightened inflammatory response or contribute to the dysregulation of thrombocytopoiesis (or megakaryocytopoiesis in mammals). The enhanced inflammation in the *ankrd26^ku6^* zebrafish might also contribute to the increased rate of thrombocyte adhesion and aggregation or thrombus formation under flow in *ankrd26^ku6^* zebrafish compared with wt controls and explain how overexpression of Ankrd26 plays a synergic role in modulating the phenotype in the zebrafish model of TTP. Future studies to dissect the functional relevance of NINJ1 in *ANKRD26* mutation-associated thrombocytopenia could shed new light on its broader implications for other hematological and inflammatory disorders.

In our proteomic study, we found marked upregulation of Lyz, Lect2l, Npsnl, Mpx and Sult2st1, along with significant downregulation of Dnttip2 and C3a.6, in the mature thrombocytes of zebrafish carrying *ankrd26^ku6^* mutation, compared with those in the wt controls. These elevated proteins are known to participate in the processes of innate immunity (Rice et al., 1987), chronic inflammation (Idler et al., 2024) and thrombocyte reactivity. Microfluidic assay demonstrated the enhanced rate of thrombocyte adhesion and accumulation onto a collagen surface under arterial flow following perfusion of the whole blood from zebrafish carrying *ankrd26^ku6^* compared with the blood from wt controls, suggestive of an enhanced hemostatic function of thrombocytes/von Willebrand factor despite the relatively low thrombocyte counts in these fish. The participation of ANKRD26 in modulation of the TTP disease process is further supported by significantly elevated ANKRD26 levels in platelets and plasma of patients with iTTP compared with those in unaffected controls. This increased expression is not inherited but acquired, likely resulting from the increased expression of protein arginine methyltransferase (PRMT1), which has been shown to be upregulated in many inflammatory diseases (Nicholson et al., 2009; Wei et al., 2014; Zheng et al., 2023). PRMT1 overexpression can modulate ADAMTS13 function and regulate ANKRD26 expression (L. Zheng, unpublished data), which enhances platelet agglutination, suppresses proplatelet production, and modulates inflammatory responses and disease severity, such as that of TTP and the rate of platelet recovery following standard of care therapy.

There are several limitations to acknowledge. First, although we observed increased *ankrd26* mRNA and protein levels in *ankrd26^ku6^* zebrafish, the exact mechanism by which the 2 bp deletion in the 5'-UTR leads to gene overexpression remains unclear. Our data suggest that this effect is not due to altered Runx1 or Fli1 transcription factor binding, as the mutated region lacks known binding sites. This indicates the presence of alternative regulatory mechanisms, which warrant further investigation. Second, although we observed hematological malignancy in *ankrd26^ku6^*;*nacre* zebrafish, the precise contribution of the *nacre* mutation and the mechanistic link to malignant transformation have yet to be fully delineated. Comprehensive genomic and microenvironmental analyses may be necessary to elucidate this interaction.

We conclude that we have established the first vertebrate model of THC2 using CRISPR/Cas9 by creating a small deletion mutation in the 5′-UTR of *ankrd26*. *ankrd26^ku6^* zebrafish develop spontaneous thrombocytopenia, which recapitulates the clinical phenotypes of THC2. Additionally, zebrafish carrying a heterozygous *ankrd26* mutation (*ankrd26^ku6/+^*) on top of *adamts13^−/−^* background develop more severe thrombocytopenia than those carrying a single gene mutation alone. Young thrombocytes from *ankrd26^ku6^* zebrafish exhibit significant upregulation of Ninj1. Patients with iTTP show increased levels of ANKRD26 in platelets and plasma. Our findings could facilitate the design of a novel targeted therapeutic for hereditary and acquired thrombocytopenia and other thromboinflammatory disorders associated with ANKRD26 abnormalities.

## MATERIALS AND METHODS

### Generation of mutations in the 5′-UTR of *ankrd26* in zebrafish by CRISPR/Cas9

Zebrafish (*Danio rerio*) were used according to the protocol approved by the Institutional Animal Care and Use Committee (IACUC; #2020-2572) of The University of Kansas Medical Center. Zebrafish *ankrd26* (NCBI accession: XP_021326262; UniProtKB/TrEMBL: A0A8M9P6L5) is situated on chromosome 25: 3573079-3647269. Mutations at the *ankrd26* locus were generated using genome editing as previously described by our laboratory (Zheng et al., 2020). The guide RNA (gRNA) targeting the 5′-UTR in exon 2 of zebrafish *ankrd26* was designed using the CRISPR design tool (http://crispr.mit.edu/). A 69-nucleotide oligonucleotide (GCGGCCTCTAATACGACTCACTATAGG**GGGTGAACGACAGTCTG-CGGA***GTTTTAGAGCTAGAAATAGCA*), consisting of a T7 promoter (underlined), a target sequence (bold) and a gRNA scaffold (italic) was synthesized at Thermo Fisher Scientific (Waltham, MA, USA). The oligonucleotide was used to assemble a gRNA-encoding template following annealing and extension with the gRNA core sequence. The gRNA was generated using a Guide-it sgRNA transcription kit from Takara-Clontech (Mountain View, CA, USA). Cas9 mRNA was synthesized from pT3TS-nCas9n using the mMESSAGE mMACHINE T3 kit of Life Technologies (Carlsbad, CA, USA). Both the gRNA and Cas9 mRNA were purified with a MEGAclear RNA purification kit (Thermo Fisher Scientific) and dissolved in RNase-free water before use. Then, 2 nl of the final product (consisting of 12.5 pg/nl gRNA and 300 pg/nl Cas9 mRNA) was injected into one-cell-stage zebrafish embryos. Genomic DNA was extracted from a fin clip from adult F0 fish. The targeted region of the *ankrd26* locus was amplified by PCR with a gene-specific primer pair (forward, 5′-ttattcttagagaatgggcg-3′; reverse, 5′-ccttatccagctggtttaaa-3′). The potential mutations in F0 founders were identified by PCR, followed by T7 endonuclease I digestion (Zheng et al., 2022), which were then crossed with wt zebrafish for three successive generations: F1, F2 and F3. The F3 heterozygotes were bred to generate F4 offspring with a reduced likelihood of off-target mutations.

### RNA extraction and real-time qPCR

Total RNA was extracted and purified from pooled larvae at 5 days post-fertilization (dpf) using an RNeasyPlus Mini Kit (Qiagen, Gaithersburg, MD, USA). The cDNA was then synthesized using a PrimeScript™ 1st strand cDNA Synthesis Kit (Takara, San Jose, CA, USA). Real-time qPCR was performed on a QuantStudio 7 Flex Real-Time PCR System with PowerTrack™ SYBR Green Master Mix reagents (Thermo Fisher Scientific).

### Western blot analysis

Pooled zebrafish larvae (*n*=10, per group) at 5 dpf were used for total protein extraction. Following denaturization (100°C, 5 min) with 1× sample buffer [50 mM Tris-HCl, pH 7.5, 0.1 M dithiothreitol (DTT), 70 mM sodium dodecyl sulfate (SDS), 1.5 mM Bromophenol Blue and 1.0 M glycerol] the proteins were separated by 10% SDS-polyacrylamide gel electrophoresis. The proteins were then transferred to a polyvinylidene fluoride membrane, which was blocked with a Tris-buffered saline containing 1% casein. A zebrafish-specific anti-Ankrd26 antibody (1:500), custom made by ABclonal (Woburn, MA, USA) using the protein fragment K55-Y219aa of zebrafish Ankrd26 as an immunogen and purified via an antigen-affinity chromatography, and an IRDye800-conjugated anti-rabbit IgG (1:5000) were used for detection. The fluorescent signals were collected with an Odyssey imaging system (LI-COR, Lincoln, NE, USA).

### Microfluidic shear-based assays

PPACK-anticoagulated whole-blood samples pooled from adult zebrafish (6-8 months old, *n*=10 each group) of different genotypes were perfused at 15 dyne/cm² over type I fibrillar collagen-coated surfaces using parallel microfluidic channels in the BioFlux system (Fluxion Biosciences, Oakland, CA, USA). Thrombocytes were endogenously labeled with a green fluorescent protein, and erythrocytes were labeled with red fluorescent protein. Digital images were captured every 3 s for a total of 120 s. The relative increase in fluorescent intensity as a function of time in wt and *ankrd26* mutant fish was determined offline using a Montage software as previously described (Zheng et al., 2020).

### Flow cytometry

Differentiation and quantification of erythrocytes (with only red fluorescence), young thrombocytes (with red and green fluorescence) and mature thrombocytes (with only green fluorescence) were performed using an FACS Vantage flow cytometer (BD Biosciences, San Jose, CA, USA) with count beads (Thermo Fisher Scientific) (Zheng et al., 2020).

### Blood or kidney marrow smear staining and analysis

Peripheral blood cells from adult zebrafish were fixed with absolute methanol and stained with Giemsa staining, and the cell images were obtained using a light microscope (Carl Zeiss, Göttingen, Germany) according to a method previously described (Zheng et al., 2020). Additionally, zebrafish kidney marrow cells were prepared and stained according to a protocol published in the literature (Ma et al., 2011).

### Histological and immunohistochemical analyses

The whole fish was fixed with 4% paraformaldehyde in phosphate buffered saline (PBS) and embedded in paraffin. Thin (6 µm) sections were prepared using a microtome for Hematoxylin and Eosin staining and immunohistochemistry. An anti-zebrafish Ankrd26 IgG (ABclonal; 1:300) was used for immunohistochemistry as previously described (Zheng et al., 2020). Additionally, human peripheral blood smears were fixed with absolute methanol and incubated with APC-conjugated anti-human CD41 antibody (Fisher Scientific; 1:200) and FITC-conjugated anti-human ANKRD26 antibody (Bioss Antibodies, Woburn, MA, USA; 1:100), followed by 4′,6-diamidino-2-phenylindole (DAPI) for nuclear staining.

### Blood cell sorting and tandem mass tag-multiplex labeling-based quantitative proteomics

Zebrafish blood was collected and anticoagulated with 5 mM EDTA after tail amputation under anesthesia. Young and mature thrombocytes were sorted from pooled zebrafish whole blood (*n*=10) using an FACS Aria cell sorter (BD Biosciences) based on fluorescent protein expression as previously described (Zheng et al., 2020). Three biological replicates were included for proteomic analysis. Briefly, cell pellets were lysed in lysis buffer containing 2% SDS and sonicated using a Sonic Dismembrator (Thermo Fisher Scientific). Protein concentrations were determined by the BCA method. An equal amount (20 µg) of total protein from each of the 18 samples was processed for trypsin digestion, followed by TMT-multiplex labeling using one TMT-18plex set (Thermo Fisher Scientific). The labeled peptides were combined and dried using a Speed-Vac (Thermo Fisher Scientific). The dried labeled peptides were then reconstituted in 20 µl of 10 mM triethylammonium bicarbonate (TEAB). To reduce sample complexity, fractionation of the TMT-labeled peptide mixture was carried out using basic reversed-phase ultra-high-performance liquid chromatography (UHPLC) with an AdvanceBio Column (2.7 µm, 2.1×250 mm; Agilent Technologies, Santa Clara, CA, USA) at Solvent A (10 mM TEAB, pH 8.0) and an UHPLC 1290 system (Agilent Technologies). The separation was performed by running a gradient of Solvent B (10 mM TEAB, pH 8.0, 90% acetonitrile) and Solvent A (10 mM TEAB, pH 8.0) at a flow rate of 250 µl/min. Eluted fractions were collected into a 96-well plate using a 1260 series auto-sample fraction collector (Agilent Technologies). The 96 fractions were subsequently consolidated into 24 fractions based on collection time for liquid chromatography–tandem mass spectrometry analysis. The analysis was conducted using an Exploris 240 Orbitrap Mass Spectrometer (Thermo Fisher Scientific) coupled with a Dionex UltiMate 3000 RSLCnano System (Thermo Fisher Scientific). The set of 24 mass spectrometry raw data files acquired from analysis of the 24 fractions was searched against the *Danio rerio* protein sequences database obtained from the UniProtKB website using Proteome Discoverer 2.4 software (Thermo Fisher Scientific, San Jose, CA, USA) based on the SEQUEST and percolator algorithms. The false positive discovery rate was set to 1%. Relative protein abundance was calculated using the ratio of abundance determined by the TMT-tags. A normalization of the ratio (relative abundance) was conducted using the summed reporter ion intensities. GO enrichment analysis was performed using the PANTHER Classification System (Mi et al., 2019).

### Quantitation of ANKRD26 protein in patients with iTTP

The cellular ANKRD26 levels in the plasma and platelets from patients with iTTP and unaffected controls were determined by western blot analysis using the polyclonal anti-ANKRD26 IgG (ABclonal; 1:1000). Plasma levels of ANKRD26 proteins were determined by enzyme-linked immunosorbent assay (AFG Scientific, Northbrook, IL, USA).

### Statistical analysis

All data are presented as the mean±s.e.m. from multiple independently repeated experiments unless specified in the figure legends. Mann–Whitney *U*-test was used to determine the statistical significance of differences between two groups; Kruskal–Wallis one-way analysis of variance (ANOVA) was used to determine the statistical significance of differences among three groups or more. All statistical analyses were conducted using Prism 10 (GraphPad Software, La Jolla, CA, USA).

## Supplementary Material

10.1242/dmm.052222_sup1 Supplementary information

Table S1. Proteomic analysis of *ankrd26^ku6^* and wt young thrombocytes.

Table S2. Proteomic analysis of *ankrd26^ku6^* and wt mature thrombocytes.
