# Supplementary material for: Modeling ANKRD26 5′-UTR mutation-related thrombocytopenia
Source: Dis Model Mech. 2025 Apr 28;18(4):dmm052222. doi: 10.1242/dmm.052222 (PMC12067082; doi:10.1242/dmm.052222)
Supplement: Supplementary information [file dmm-18-052222-s1.pdf]

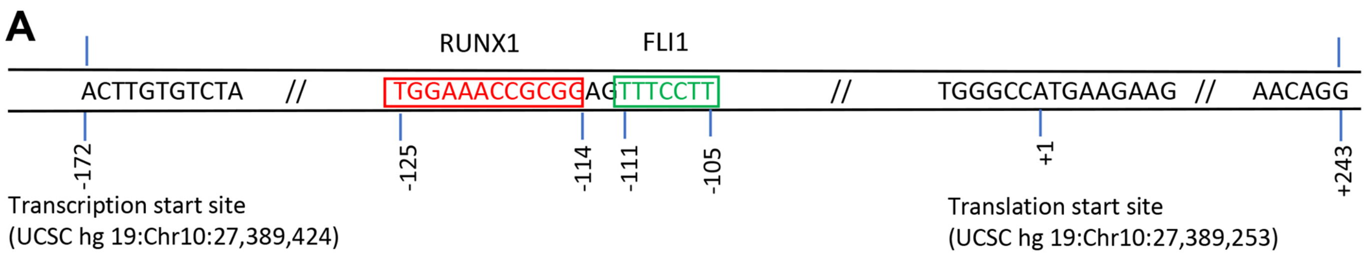

B

```
                   10        20        30        40        50        60        70        80        90       100
           ....|....|....|....|....|....|....|....|....|....|....|....|....|....|....|....|....|....|....|....|
human      ----------------------------------------------------------GTGTCTAGC-GGATCGCTTGCTTGGTAACCCGGAGGGAGAGA
mouse      --------------------GTGAGCATGCGCGGCAGCAAGAAGGCGCGGGTTGCTA..T.GC...-TC.GCTTGGC.A..AT.GTG.TTT.AAA.CCTG
zebrafish  CGTTCCGTGTGAATGCAGAGGAAAAAACAGCGAGTTCCGTGCTCGTAGAGTAGTCGAA.T.G.GATATTA.A.ATACAT.CAAC.CGTTAA.AT.TATTT

                  110       120       130       140       150       160       170       180       190       200
           ....|....|....|....|....|....|....|....|....|....|....|....|....|....|....|....|....|....|....|....|
human      TTGGAAACCGCGGAGTTTCCTTTGGGAGGCT-------GCGGCCAGCCGGGGCTGACTTGTTATGTTGGGCTCCGGAGGC----CGTTAAGA-GCCGA--
mouse      CC..GCCAT..A.C..C.TTCCC..TG..A.-------C.C.TGC.T.TA...C.G...T.C.ATAA.T..C.TA.CT..GTTGT...GTC.-...A.CG
zebrafish  AAAT..T...TTCT....T..CAACT.TTA.TTATTTG..ACT..ATTTAAAA...A..A...G.A.CA.GATGA.C.T.ATGCT....CC.T.GTA.CA

                  210       220       230       240       250       260       270       280       290       300
           ....|....|....|....|....|....|....|....|....|....|....|....|....|....|....|....|....|....|....|....|
human      ------GAGAGACATGAGGTGTCTCTGAAGCCCGGTCGCCTGGGCC------------------------------------------------------
mouse      TTGC--.C.G.GA.GA.....C..G...G.GG..-.AC...CT...------------------------------------------------------
zebrafish  GAAGCA.CT..CTTGCTA.C...GTAA.C.G.TAA...TT..A..AGCATTTATGTGTCATACTCGATAACGGAGATCATTTAAACATATTTAATGTTTA

                  310       320       330       340       350       360       370       380       390       400
           ....|....|....|....|....|....|....|....|....|....|....|....|....|....|....|....|....|....|....|....|
human      ----------------------------------------------------------------------------------------------------
mouse      ----------------------------------------------------------------------------------------------------
zebrafish  TTTATTTTTTTAAAACATCAGTGATAGACGCCCTGCAGCGAAACCACGATGATTTATTCTTAGAGAATGGGCGAGACATGCGAGGCGATCAGTACCTGAG

                  410       420       430       440
           ....|....|....|....|....|....|....|....|....|..
human      -----------------------------------------------
mouse      -----------------------------------------------
zebrafish  CCTCAGGTGAACGACAGTCTGCGGATGGAGATGTGCCGTCAGCGACC
```

Fig. S1. Putative RUNX1 and FLI1 binding motifs in 5'UTR of ANKRD26 are not conserved in mouse and zebrafish. **A**, Schematic representation of 5'UTR of human *ANKRD26* gene indicates RUNX1 (red) and FLI1 (green) binding sequences. **B**, Sequence alignment of 5'UTR of Ankrd26 in human (NM_014915), mouse (NM_001081112) and zebrafish (XM_021470586). RUNX1 and Fli1 binding sites in human *ANKRD26* are indicated by red and green box, respectively. Black box indicates the 2 nucleotides deletion mutation created in the zebrafish *ankrd26* gene. Dots represent same nucleotides and dashes represent nucleotide gaps among species.

Disease Models & Mechanisms • Supplementary information

Table S1. Proteomic analysis of *ankrd26^{ku6}* and wt young thrombocytes.

Available for download at
https://journals.biologists.com/dmm/article-lookup/doi/10.1242/dmm.052222#supplementary-data

Table S2. Proteomic analysis of *ankrd26^{ku6}* and wt mature thrombocytes.

Available for download at
https://journals.biologists.com/dmm/article-lookup/doi/10.1242/dmm.052222#supplementary-data
